# Endometrial Mesenchymal Stem/Stromal Cells Modulate the Macrophage Response to Implanted Polyamide/Gelatin Composite Mesh in Immunocompromised and Immunocompetent Mice

**DOI:** 10.1038/s41598-018-24919-6

**Published:** 2018-04-26

**Authors:** S. Darzi, J. A. Deane, C. A. Nold, S. E. Edwards, D. J. Gough, S. Mukherjee, S. Gurung, K. S. Tan, A. V. Vashi, J. A. Werkmeister, C. E. Gargett

**Affiliations:** 1 The Ritchie Centre, Hudson Institute of Medical Research, 27–31 Wright Street, Clayton, Victoria 3168 Australia; 2 Department of Obstetrics and Gynaecology, Monash University, Clayton, Victoria 3168 Australia; 3 CSIRO Manufacturing, Bayview Avenue, Clayton, Victoria 3169 Australia

## Abstract

The immunomodulatory properties of human endometrial mesenchymal stem cells (eMSC) have not been well characterised. Initial studies showed that eMSC modulated the chronic inflammatory response to a non-degradable polyamide/gelatin mesh in a xenogeneic rat skin wound repair model, but the mechanism remains unclear. In this study, we investigated the immunomodulatory effect of eMSC on the macrophage response to polyamide/gelatin composite mesh in an abdominal subcutaneous wound repair model in C57BL6 immunocompetent and NSG (NOD-Scid-IL2Rgamma^*null*^) immunocompromised mice to determine whether responses differed in the absence of an adaptive immune system and NK cells. mCherry lentivirus-labelled eMSC persisted longer in NSG mice, inducing longer term paracrine effects. Inclusion of eMSC in the mesh reduced inflammatory cytokine (Il-1β, Tnfα) secretion, and in C57BL6 mice reduced CCR7^+^ M1 macrophages surrounding the mesh on day 3 and increased M2 macrophage marker mRNA (*Arg1*, *Mrc1, Il10*) expression at days 3 and 7. In NSG mice, these effects were delayed and only observed at days 7 and 30 in comparison with controls implanted with mesh alone. These results show that the differences in the immune status in the two animals directly affect the survival of xenogeneic eMSC which leads to differences in the short-term and long-term macrophage responses to implanted meshes.

## Introduction

Tissue engineering (TE) combines cells and materials to create implants that improve repair of injured tissues^1–3^. Huge advances in tissue engineering have occurred over the past two decades with the development of new biomaterials and the use of various adult stem cells, MSC in particular. Tissue engineering has great potential for use in women’s urogynaecological health including treatment of stress urinary incontinence (SUI) and pelvic organ prolapse (POP)^4–6^.

Pelvic Organ Prolapse is the herniation of pelvic organs into the vagina. Symptoms include voiding, bowel and sexual dysfunction and incontinence^7^. POP affects more than 25% of all women and 19% of women undergo reconstructive surgical treatment, often involving surgical mesh to provide mechanical support to damaged tissue^8,9^. Monofilament polypropylene mesh with a large pore size allows for greater neo-tissue ingrowth and is the most common type of mesh used in POP surgery^10^. However, vaginal insertion of mesh has resulted in complications in approximately 10% of women^11^ including mesh erosion, mesh contracture, infection and pain^12^. The use of tissue engineering constructs may reduce these adverse effects.

Adult mesenchymal stem cells have been used as a cell-based therapy in tissue engineering applications to deliver reparative cells to damaged tissue sites to effect tissue repair and regeneration^13^. A number of tissue engineering approaches using bone marrow mesenchymal stem cells (bmMSC) have been explored in abdominal wall hernia repair^14,15^. More commonly, bmMSC have been administered intravenously, where they home to damaged and injured tissues and exert anti-inflammatory and immunomodulatory effects, without tissue incorporation^16^. Human endometrial MSC (eMSC) are a recently identified MSC type that are easily accessible by a minimally invasive office-based biopsy procedure without anaesthesia^17,18^. Clonogenic eMSC can be purified using co-expression of CD140b and CD146 with a cell sorter or by a single marker, SUSD2 (recognised by the W5C5 antibody) using magnetic bead sorting^19,20^. These perivascular eMSC fulfil the International Society for Cellular Therapies minimal MSC criteria, are highly proliferative, self-renew *in vitro* and reconstitute stromal tissue *in vivo*^19,21^.

We are developing and evaluating a new type of mesh for potential clinical use in POP treatment. In particular, we fabricated a new tissue engineered construct comprising a novel polyamide knitted mesh coated with stabilised gelatin (PA + G)^22,23^. Comparison of the structural characteristic and mechanical properties of the mesh with three commercial PP meshes showed that our PA + G mesh was less stiff and had lower bending rigidity, more desirable properties for POP surgical repair. We have also shown the efficacy of using eMSC in PA + G mesh^23^ to deliver eMSC in a small animal model of wound repair^24^. In this xenogeneic model, human eMSC exerted a paracrine effect promoting wound healing, angiogenesis and neo tissue formation. Seeding the mesh with eMSC enhanced the biocompatibility of the mesh by reducing chronic inflammation and promoting the deposition of crimped physiological collagen around mesh filaments resulting in reduced stiffness of the mesh-tissue complex in the long term^25^. Thus, eMSC-seeded PA + G mesh may provide an alternative treatment option for POP^17^.

One of the main concerns in tissue engineering is the host response to biomaterials^26^. Macrophages play a key role in host response to biomaterials^27,28^ by secreting cytokines and chemokines that impact on tissue repair^29^. Understanding the exact role of macrophages in host response to implanted biomaterials and the factors that they may secrete to regulate this response is important.

Several lines of evidence have demonstrated crosstalk between macrophages and MSC. Macrophages co-cultured with bmMSC expressed higher levels of CD206, the mannose receptor, secreted anti-inflammatory IL10 and reduced levels of Tumor Necrosis Factor-α (TNF-α) in the microenvironment^30^. In a sepsis mouse model, infusion of mouse bmMSC decreased lethality, and the protective effect of the MSC was eliminated by macrophage depletion or by the administration of Il10 neutralizing antibodies^31^. Il10 produced by M2 macrophages blocked excessive neutrophil infiltration into the injured tissue and prevented further damage^31^. Similarly, in an endotoxin-induced lung injury model, intrapulmonary delivery of mouse bmMSC decreased the production of Tnfα and Chemokine Ligand 2 (CXCL2) and increased alveolar macrophage secretion of Il10^32^.

To further elucidate the early immune responses to eMSC-seeded PA + G mesh, the aim of the current study was to determine whether eMSC alter macrophage polarization from an M1 inflammatory to an M2 wound healing phenotype in response to implanted mesh in both early and later stages of the host response. We compared two mouse models; immunocompromised (NSG) and immunocompetent (C57BL6) mice. NSG mice lack an adaptive immune system and NK cells, and have a defective innate immunity associated with functionally immature macrophages and deficiencies in several cytokine signalling pathways^33^. We compared the anti-inflammatory and immunomodulatory effects of eMSC on macrophage responses between mouse strains with intact (C57BL6) and defective (NSG) immune systems. We also assessed whether xenogeneic, heterologous eMSC persisted longer in the absence of an adaptive and deficient innate immune system.

## Results

### eMSC transduction and survival ***in vivo*** on PA + G mesh

**Figure 1:**
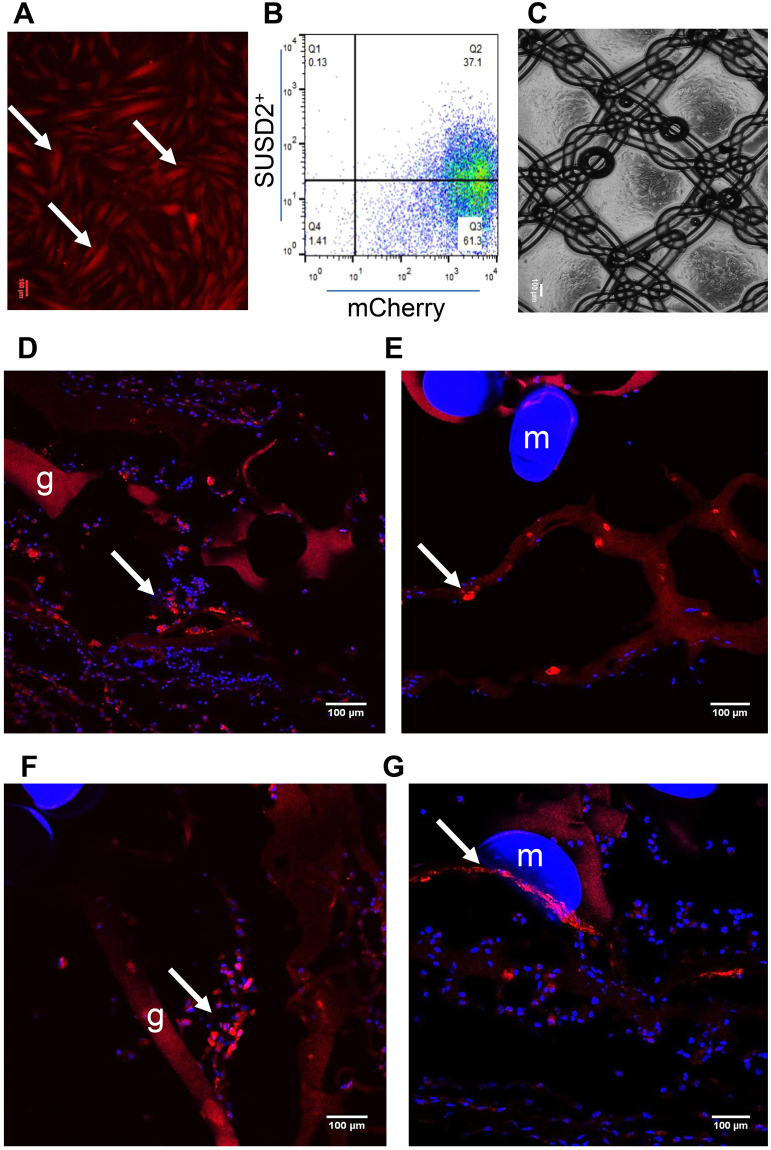
eMSC transduction and survival of eMSC on PA + G mesh in NSG mice. (**A**) cultured mCherry transduced eMSC showing red fluorescence, (**B**) more than 95% of transduced and cultured eMSC were mCherry^+^ by flow cytometry and about 40% of this population were SUSD2^+^. Representative trace of n = 6 patient samples, (**C**) PA + G mesh seeded and cultured with eMSC. (**D**,**E**) mCherry^+^ eMSC were observed 3 and (**F**,**G**) 7 days post-implantation around the mesh filaments in immunocompromised NSG mice. Arrows, representative mCherry^+^ eMSC; m, mesh filament; g, gelatin. Scale Bars 100 µm.

In order to track the cells *in vivo*, eMSCs purified by SUSD2 magnetic bead sorting were transduced with mCherry lentiviral vector and the SUSD2^+^mCherry^+^ cells were sorted and cultured in DMEM medium containing 10% FBS. More than 95% of cultured eMSCs were mCherry^+^ (Fig. 1A and B) and around 40% of this population were SUSD2^+^. Double positive cells were sorted, expanded and seeded on PA + G mesh (Fig. 1C). The persistence of implanted eMSC was assessed by fluorescence microscopy. Three days after mesh implantation, mCherry^+^ eMSC were found around the mesh filaments and in the gelatin layer in NSG mice (Fig. 1D and E). Fewer mCherry^+^ cells persisted after 7 days (Fig. 1F and G). No mCherry^+^ eMSC were detected in C57BL6 mice after 3 or 7 days implantation.

H&E staining showed the degree of cellularity around the implanted mesh at the later time points (days 14 and 30) in both NSG and C57BL6 mice of the eMSC/mesh and mesh control groups. The gelatin layer coated on the top of the mesh gradually degraded with the time. Visually, there was a higher number of infiltrated cells in immunocompetent mice compared with immunocompromised mice. (Supplementary Fig. 1A–H).

### eMSC induce macrophage polarization

**Figure 2:**
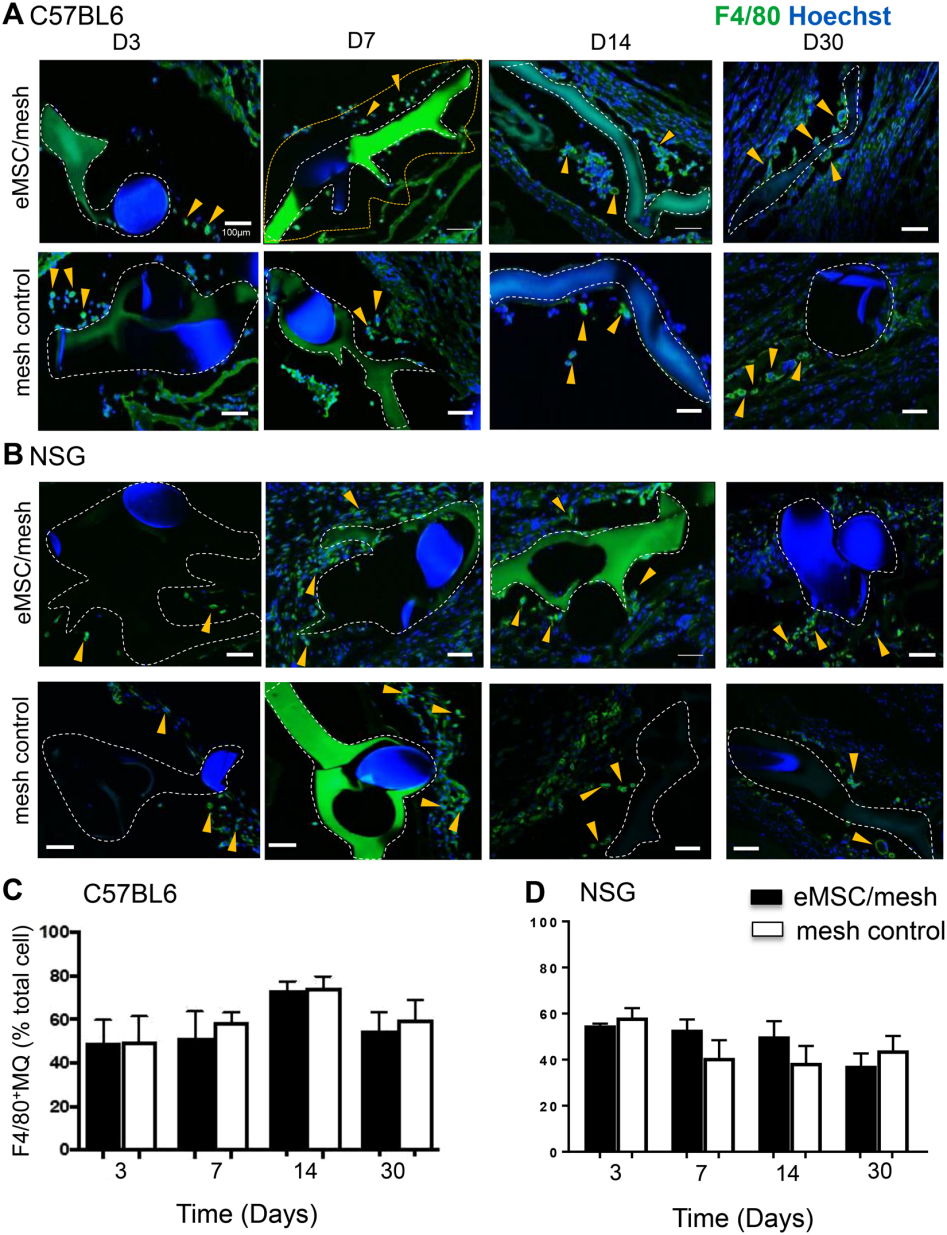
Macrophages surround implanted mesh with or without eMSC. Representative images of F4/80 immunostaining in (**A**) C57BL/6 mice implanted with eMSC/PA + G mesh (upper panel) and PA + G mesh control (lower panel) for 3–30 days, and similarly implanted in (**B**) NSG mice. Macrophages (F4/80^+^, green) are shown as percentage of total nucleated cells in the first 100 µm increment around mesh filament in (**C**) C57BL/6 mice and in (**D**) NSG mice. Data are mean ± SEM of n = 6 animals/group and analyzed by two-way ANOVA. m, mesh, g, gelatin, white dashed lines, outline of PA + G mesh, yellow dotted lines show the 100 µm increment around mesh analyzed eg in (A, D7). Arrowheads, representative F4/80 macrophages both within and outside the 100 µm increment around PA + G mesh. Scale bars 100 µm.

Image analysis quantification within the first 100 µm increment around individual mesh filaments showed that the percentage of total macrophages (F4/80^+^) relative to Hoechst-stained nucleated cells in C57BL6 and NSG mice was relatively constant from day 3 to 30 in both groups; PA + G mesh seeded with and without eMSC (Fig. 2A and C).

**Figure 3:**
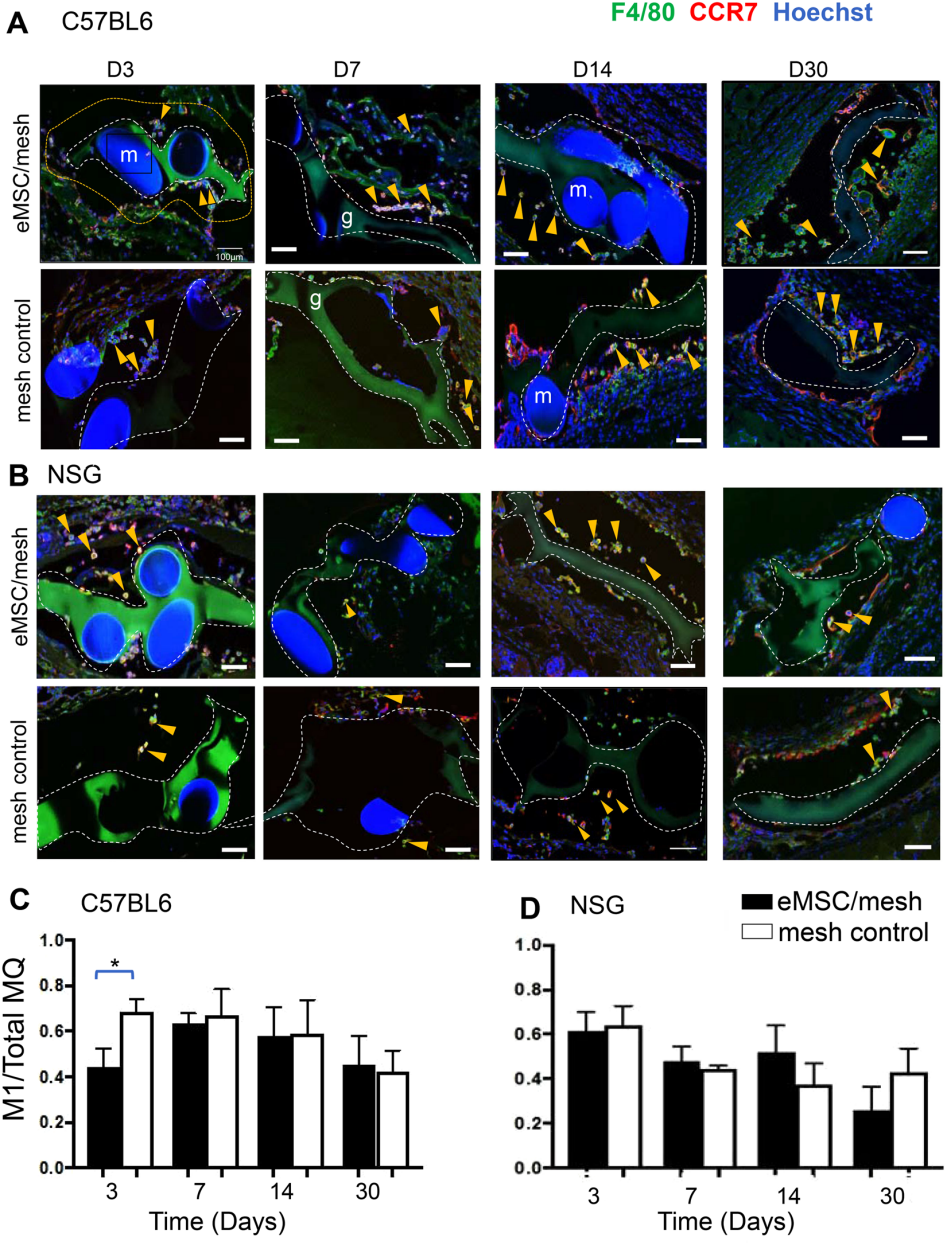
M1 macrophage quantification around implanted mesh in C57BL/6 and NSG mice. CCR7 M1 macrophages (red) co-localized (yellow) with the pan F4/80 macrophage marker (green) around implanted mesh in mesh/eMSC and mesh control groups in (**A**) C57BL6 mice and in (**B**) NSG mice. The ratio of CCR7^+^ macrophages to total F4/80 macrophages (MQ) in the first 100 µm increment around mesh filaments in (**C**) C57BL6 mice and in (**D**) NSG mice. Data are mean ± SEM of n = 6 animals/group and were analyzed by two-way ANOVA. *P < 0.05. Arrows show representative CCR7^+^F4/80^+^ macrophages both within and outside the 100 µm increment around PA + G mesh, m, mesh filament; g, gelatin. Scale Bars 100 µm.

To determine the phenotype of F4/80^+^ macrophages accumulating around the eMSC/PA + G (eMSC/mesh) and non-seeded PA + G (mesh control), dual color immunofluorescence staining was performed to quantify M1 (CCR7/F4/80) and M2 (CD206/F4/80) macrophages in sections from C57BL6 and NSG mice at 3, 7, 14 and 30 days. In mesh seeded with eMSCs, the number of M1 expressing macrophages was significantly reduced in comparison to the control implants at day 3 in immune intact (C57BL6) mice (Fig. 3A and C). No significant differences between eMSC/mesh and mesh controls were observed at the later time points in C57BL6 mice. In immunocompromised mice (NSG), there was no difference in M1 macrophages from 3 to 30 days between the eMSC/mesh and mesh control groups (Fig. 3B and D).

**Figure 4:**
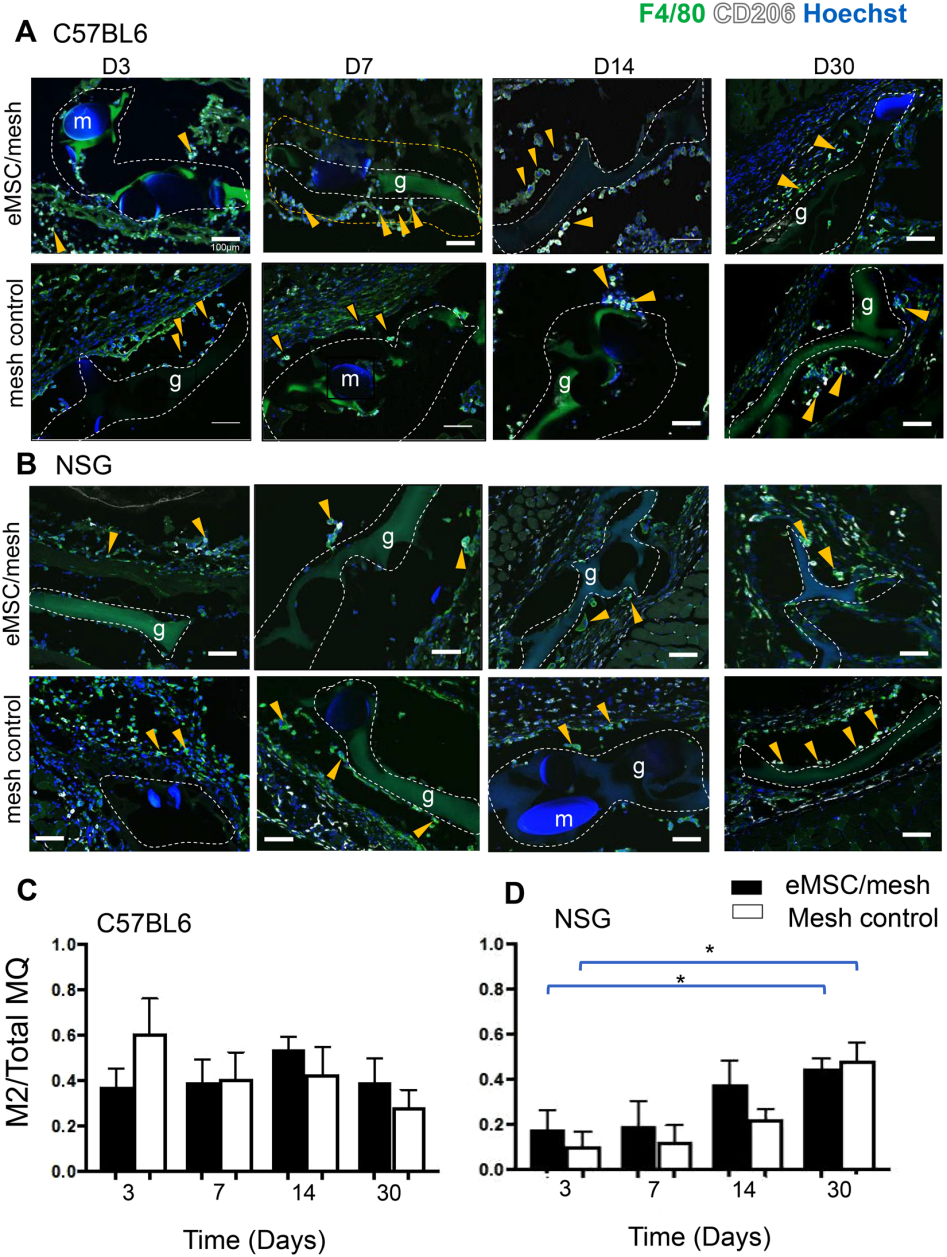
M2 macrophage quantification around implanted mesh in C57BL/6 and NSG mice. CD206 M2 macrophage (white) co-localized with the pan macrophage F4/80 marker (green) in (**A**) C57BL/6 mice and in (**B**) NSG mice. The ratio of CD206^+^ macrophages to total F4/80 macrophages (MQ) in the first 100 µm increment around mesh filaments in (**C**) C57BL/6 and in (**D**) NSG mice. Data are mean ± SEM of n = 6 animals/group and were analyzed by two-way ANOVA. *P < 0.05. Arrows show representative CD206^+^F4/80^+^ macrophages within and outside the 100 µm increment around PA + G mesh, m, mesh filament; g, gelatin. Scale Bars 100 µm.

We next used CD206 to investigate M2 macrophages, a hallmark cell associated with tissue regeneration and healing, around the implanted mesh filaments. There was no significant difference in the proportion of CD206-expressing macrophages between eMSC/mesh and mesh control groups in immunocompetent mice at any time point. (Fig. 4A and C). In the immunocompromised NSG mice, the M2 macrophage proportion gradually increased with time in both mesh control and eMSC/mesh groups with significantly more M2 macrophages present at day 30 compared with day 3, (P < 0.05) (Fig. 4B and D). However, there was no difference between the density of M2 macrophages around mesh filaments whether eMSC were present or not. The M2/M1 ratio was higher in eMSC/mesh compared to mesh control in NSG and C57BL6 mice. There was a non- significant trend for the M2/M1 ratio to increase from day 3 to day 30 in NSG mice in either eMSC/mesh and mesh control groups (Supplementary Fig. 2A and B).

### eMSC modulate inflammatory cytokine mRNA expression

**Figure 5:**
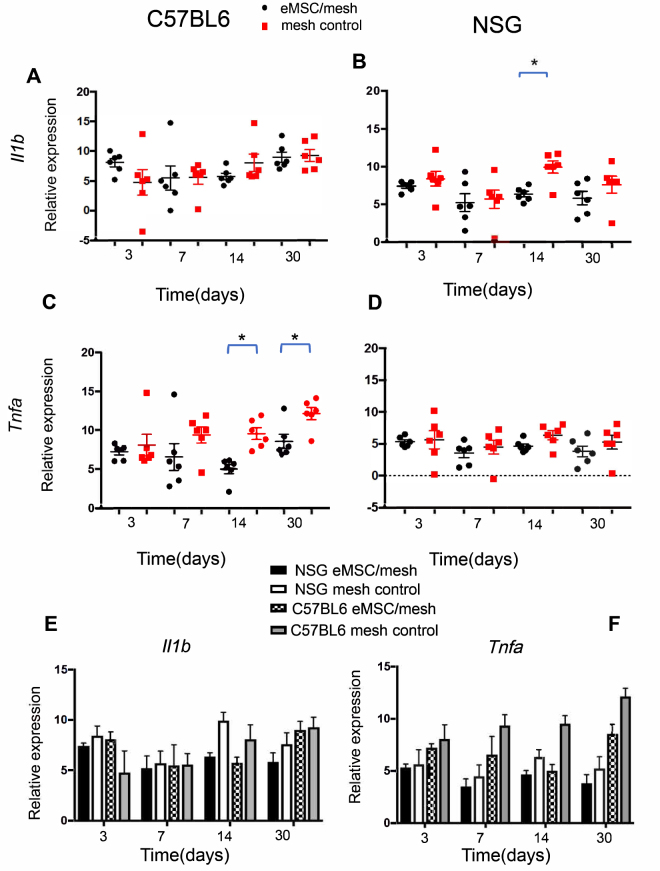
mRNA expression of M1 macrophage markers by Quantitative PCR. *Il1b* and *Tnfa* mRNA expression in eMSC/mesh and mesh control groups in (**A**,**C**) C57BL6 and in (**B**,**D**) NSG mice. Data were analyzed using two-way ANOVA. (**E**,**F**) total comparison of *Il1b* and *Tnfa* mRNA expression between the two types of mice, two groups and four time-points using 3 way ANOVA. Error bars mean +/− SEM. *P < 0.05.

To further assess the inflammatory response to the implanted mesh, the mRNA expression of the inflammatory cytokines, *Il1b* and *Tnfa* was quantified by q-PCR. In C57BL6 mice, no differences were observed in *Il1b* mRNA expression between the groups at any time-point (Fig. 5A). *Tnfa* expression was significantly reduced in eMSC/mesh group at days 14 (P = 0.008) and 30 (P = 0.01) compared with mesh control group (Fig. 5C).

In NSG mice, there was a significant decrease in *Il1b* gene expression eMSC/mesh group compared with the mesh control group at day 14 (P = 0.0023) (Fig. 5B) with no differences at the other time points. No significant difference was observed in *Tnfa* gene expression between eMSC/mesh and mesh control groups at any time point (Fig. 5D). Comparison between immunocompetent and immunocompromised mice showed no significant difference in *Il1b* and *Tnfa* gene expression at any of the four time-points (Fig. 5E and F).

### eMSC modulate inflammatory cytokine secretion

**Figure 6:**
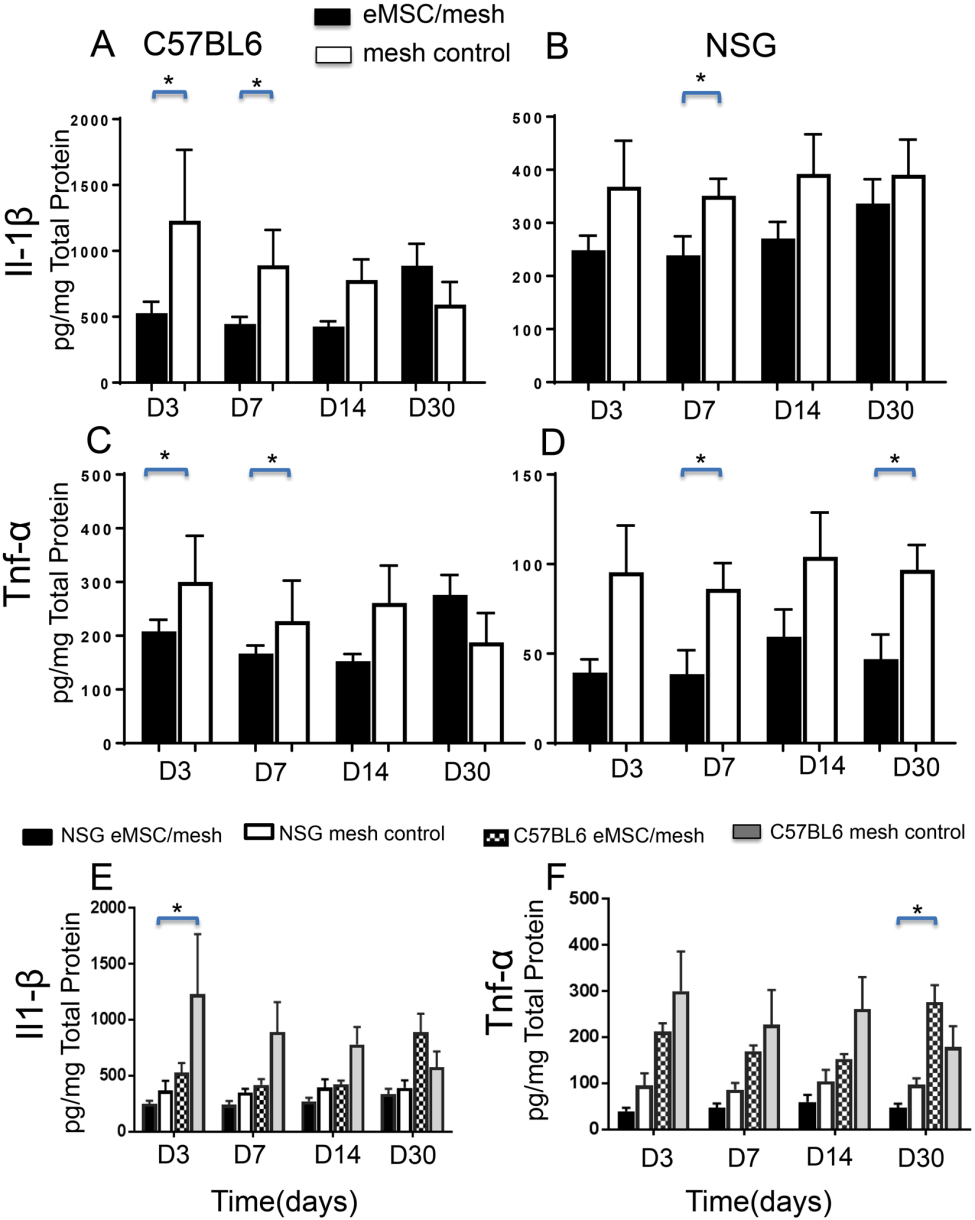
Inflammatory M1 macrophage cytokine secretion assayed by ELISA. Il-1β and Tnf-α secretion in eMSC/mesh and mesh control group implants in (**A**,**C**) C57BL/6 mice and in (**B**,**D**) NSG mice. Data were analyzed using two-way ANOVA. (**E**,**F**) comparison of cytokine expression between the two mouse models for eMSC/mesh and mesh control and four time-points using 3 way ANOVA. Data are mean +/− SEM of n = 6 animals/group. *P < 0.05.

As the qPCR quantification of inflammatory cytokine mRNA expression was not conclusive, the secretion of Il-1β and Tnf-α protein was measured in explanted tissue lysates from C57BL6 and NSG mice. In immunocompetent mice, Il-1β and Tnf-α were reduced at the early time points (days 3 and 7) in the eMSC/mesh group compared with the mesh control group (Fig. 6A and C). In the NSG mice, both Il-1β and Tnf-α secretion levels were significantly reduced in the eMSC/mesh group compared with the mesh control group at day 7 for both inflammatory cytokines, and also at day 30 for Tnf-α (Fig. 6B and D). The cytokine levels were noticeably lower in the tissues from immunocompromised NSG mice than in immunocompetent C57BL6 mice (Fig. 6E and F). There was significantly less Il-1β secretion in the mesh control group of NSG mice compared to C57BL6 mice at day 3 (P < 0.05) (Fig. 6E). Tnf-α was significantly lower in NSG mice in the eMSC/mesh group compared to the same group in C57BL6 mice after 30 days implantation (P < 0.05) (Fig. 6F).

### eMSC induce the expression of M2 macrophage markers

**Figure 7:**
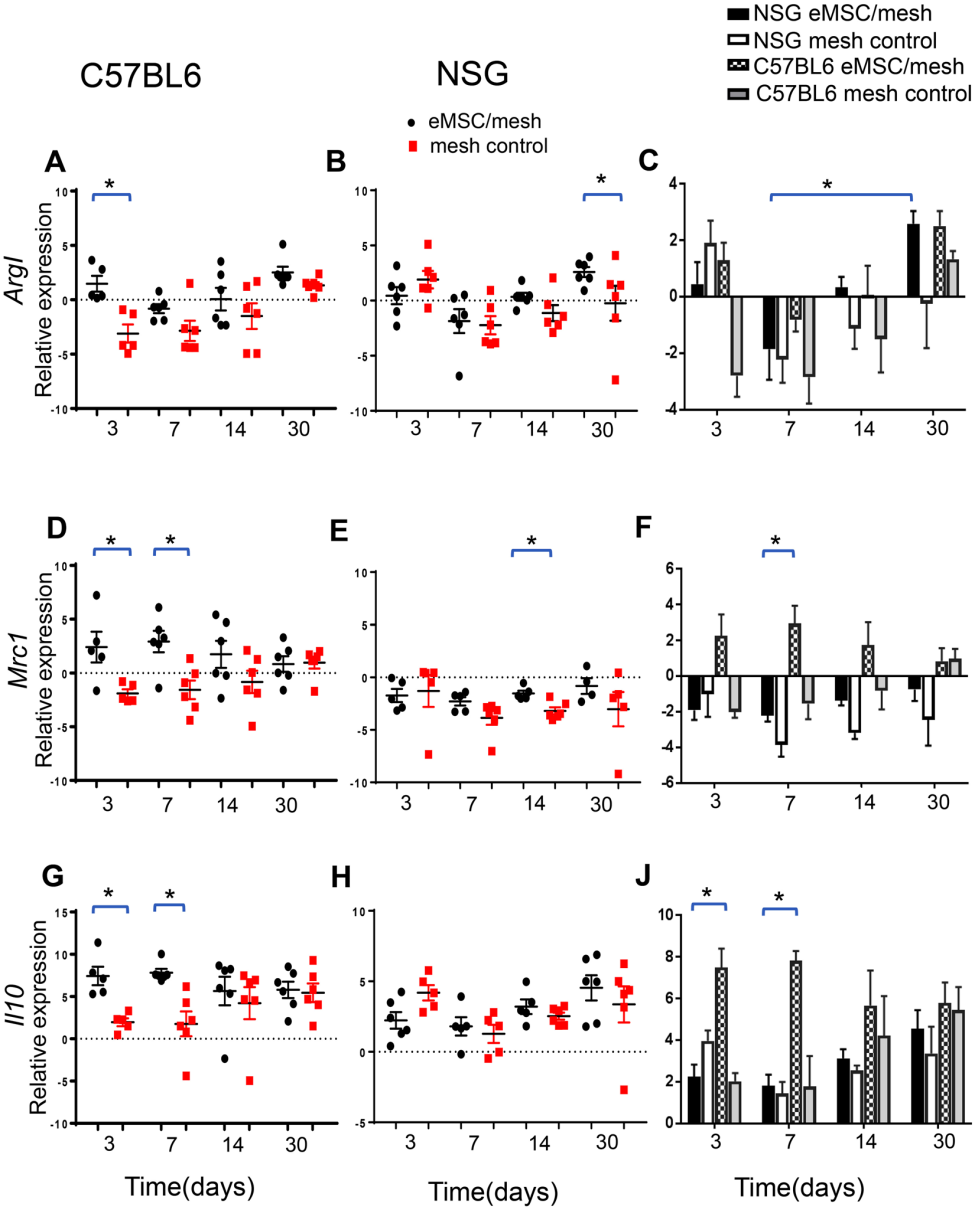
mRNA expression of M2 macrophage markers by Quantitative PCR. *ArgI*, *Mrc1* and *Il10* in eMSC/mesh and mesh control groups in (**A**,**D**,**G**) C57BL6 and (**B**,**E**,**H**) NSG mice. Data were analyzed using two-way ANOVA. (**C**,**F**,**J**) comparison of *ArgI, Mrc1* and *Il10* expression between the two types of mice, eMSC/mesh and mesh control and four time-points using 3 way ANOVA. Error bars mean +/− SEM. *P < 0.05.

We next assessed whether M2 macrophage marker mRNA expression was altered by the eMSC using q-PCR measurement of *Il*, Arginase I (*ArgI*) and Mannose Receptor (*Mrc1*). *Arg1, Mrc1* and *Il10* mRNAs were significantly higher at day 3 in the eMSC/mesh group compared to the mesh control in immune intact C57BL6 mice (Fig. 7A,D and G). Similarly, at day 7, both *Mcr1* and *Il10* were increased in the eMSC/mesh group. In immunodeficient NSG mice *ArgI* expression was significantly increased in the eMSC/mesh group compared to the mesh control group at 30 days (Fig. 7B). *Mrc1* expression was significantly higher in the eMSC/mesh group at 14 days, compared with the mesh control (Fig. 7E). Over time, *ArgI* expression increased in the eMSC/mesh group from day 7 to day 30 in NSG mice (Fig. 7C). Comparison between C57BL6 and NSG mice showed that *Mrc1* expression was significantly higher in the eMSC/mesh group in C57BL6 mic, compared with same group in NSG mice at day 7 (P = 0.03) (Fig. 7F). Similarly, *Il10* expression was significantly higher in eMSC/mesh group in C57BL6 mice, compared with NSG mice at days 3 and 7 (P = 0.001and P = 0.002, respectively) (Fig. 7J).

## Discussion

The main findings of this study are that human eMSC changed the macrophage phenotype from M1 to M2 in both immunocompromised and immune intact mouse models resulting in a reduction of inflammatory cytokine secretion and an increase in M2 macrophage marker expression. These immunomodulatory effects mediated by eMSC were delayed and weaker in immunocompromised compared to immune intact mice, likely due to the persistence of eMSC and their slower disappearance in NSG mice. It is likely that eMSC exerted these immunomodulatory effects via a paracrine mechanism in both mouse models.

The level of the macrophage infiltration around the mesh was similar in the two animal models and the presence of eMSC had minimal impact on the absolute number of infiltrating macrophages. In the C57BL6 mice, where eMSC were not readily detected even as early as 3 days after eMSC/mesh implantation, there was a significant reduction of the inflammatory CCR7^+^ M1 macrophages. This was accompanied by reduced secretion of Il-1β and Tnf-α during the first 7 days and reduced gene expression at later time-points, and upregulation of the M2 mRNA markers, *Arg1, Mrc1* and *Il10*. These findings indicate for the first time that the eMSC have significant immunomodulatory and anti-inflammatory properties *in vivo* through their effect on macrophage phenotype in immunocompetent animal models. The effect of eMSC on the macrophage response to implanted mesh was similar in NSG mice, however it was delayed and the cytokine response was blunted even though the eMSC survived longer. This was shown by the prolonged elevation of CD206 M2 macrophages and increased M2 mRNA markers, *Mrc1* and *Arg1* up to day 30, accompanied by similar and ongoing reductions of pro-inflammatory cytokine secretion and mRNA expression. This persistence of xenogeneic human eMSC in immunocompromised mice suggests that their autologous use in women, where their removal is expected to be delayed, may have greater long term beneficial effects in modulating macrophage responses to foreign mesh materials than an allogeneic source.

Macrophages play a central role in the host response to implanted biomaterials, with both M1 and M2 macrophages having essential roles and the balance of M2/M1 determining the failure or success of implantation^34^. To prevent prolonged detrimental effects of persisting M1 macrophages on implanted non-degradable biomaterials, a timely switch from a M1 to M2 macrophage phenotype can be favourable, a process facilitated by eMSCs. Our data shows there was a trend for the M2/M1 ratio to increase in NSG model, suggesting that co-transplantation of eMSC with a non-degradable biomaterial may be beneficial in reducing the chronic inflammatory response.

In this study, we quantified the effect of a recently described MSC on macrophage phenotype after mesh implantation. We showed that eMSC seeded on a synthetic composite mesh significantly reduced inflammation for up to 30 days in immunocompetent mice, despite their rapid disappearance, indicating that their modulatory effects influence the acute and chronic phase of the immune response. Other studies have also shown that injected or implanted MSC (from other sources) do not survive at the site but nevertheless limit tissue injury during their short stay through a variety of mechanisms^35,36^. In an immune intact system as in C57BL6 mice, MSC influence most immune cell types, including those mediating innate immunity; NK cells, dendritic cells, neutrophils, and adaptive immunity cells; T and B lymphocytes.^16^. Based on previous studies, it is clear that MSC regulate the inflammatory response *in vitro* and *in vivo*^37^. Our study focussed on the effect of a recently identified source of MSC derived from the endometrium (eMSC). These eMSC are an easily accessible source of adult stem cells and they do meet the minimal ISCT MSC criteria like other sources of MSCs. But they have some unique properties that we have reported on previously that includes their pericyte localisation, higher clonogenicity and higher purity when selected with the SUSD2 marker that provides an indicator of more effective stem cell function^18^. In this current study we have examined the effect of these eMSC on the macrophage response, showing that they inhibit inflammation by influencing the switching of M1 macrophages to a M2 phenotype, reducing inflammation at early and late time points. While we did not examine which eMSC mediators reduced the inflammatory response to PA + G mesh, it is possible that they operate in a similar manner to other MSC. For example, bmMSC upregulate the expression of Indoleamin2,3 dioxygenase (IDO), which induces the polarization of M1 macrophages to M2 phenotype and promote M2 macrophages secretion of IL10^16^. Another possibility is the production of PGE2 and TGF-β by eMSC, since these molecules induce M2 phenotype switching^38^. To date there are no studies on the MSC mediators of M2 switching, PGE2 or TGFβ, by eMSC. However, we found increased COX1 mRNA expression, a key enzyme involved in PGE2 synthesis, in eMSC treated with A83-01, a TGFβ-receptor inhibitor, to prevent differentiation and maintain eMSC stemness, compared with non-treated cells (Gurung S, Werkmeister J, Gargett C, un-published data) suggesting that PGE2 may be involved in eMSC modulatory effects on macrophages. Comparison of eMSC and endometrial stromal fibroblast transcriptomes also showed higher TGFβ expression in eMSC^39^. These data suggest that eMSC have the potential to function in a similar manner as other immunomodulatory MSC. Our ongoing studies are investigating these possibilities.

In NSG mice, the proportion of CD206^+^ M2 macrophages increased after 30 days in parallel with reduced levels of inflammatory cytokine secretion and mRNA expression. The lack of T, B and NK cells and defective dendritic cells (DC) and macrophages in NSG mice^40^, likely explains the lower cytokine production observed around the mesh filaments compared with C57BL6 mice. In an immunocompromised system, where longer survival of implanted stem/progenitor cells is expected, eMSC may also exert longer-term immunomodulatory effects. In a mouse model of asthma, most of the PKH2-labelled MSC injected IV into BALB/C mice were phagocytosed by macrophages and these macrophages subsequently acquired an M2 suppressive phenotype^41^. However, it is unknown whether macrophages in NSG mice phagocytose eMSC, and this needs further investigation. One of the limitations of our previous study on the paracrine effects of eMSC on mesh implantation^24,25^ was the inability to explain the late M1 to M2 switch. In this study only phenotypic CD profiling was used. Macrophages are heterogeneous, and activation and polarization into functional subsets is complex. In our current study, we have complimented the CD phenotype with quantification of more sensitive functional mediators indicative of macrophage status such as inflammatory cytokines and M2 macrophage markers. We detected genetically labelled eMSC surviving for at least 7 days in NSG mice in the current study and up to 30 days in other studies implanted under the kidney capsule in NSG mice^42^ indicating their potential to have longer term effects as demonstrated by M2 macrophage marker expression and inflammatory cytokine secretion.

Previous studies of bone marrow MSC effects on wound healing revealed that MSC secrete factors including VEGF, bFGF, IL-6 and MCP1 which accelerate wound healing and tissue regeneration by attracting macrophages^43^. We did not assess whether eMSC secreted these factors. However, our findings on the up-regulation of M2 macrophage markers in the mesh/eMSC group in the absence of eMSC persistence and their differentiation suggests paracrine effects of eMSC on macrophage polarization in NSG and C57BL6 mice. Further studies are needed to provide a better understanding of eMSC immune regulatory effects.

In summary, we have characterised some of the immunomodulatory properties of eMSCs *in vivo* for the first time. We showed that eMSC mediate inhibitory effects in both immune intact and immune compromised systems suggesting that immune intact mice are suitable for tissue engineering studies using eMSC, although the implanted cells do not persist in the latter. Our ongoing work will characterise these factors to fully understand the immunoregulatory mechanism of eMSC on innate immune cells, in particular macrophages.

## Material and Methods

### Endometrial Tissues

Endometrial biopsies were obtained from 6 women undergoing laparoscopic surgery for non-endometrial gynaecological conditions and had not taken hormonal treatment for three months before surgery. All women gave written informed consent. Our protocol was approved by the Monash Health and Monash University Human Research Ethics committees (09270B). All methods were performed in accordance with Monash Health and Monash University guidelines. Each patient sample was used to generate an individual eMSC cell line (n = 6).

### Isolation of SUSD2^+^ eMSC and Culture

Endometrial tissues were minced with scissors and digested using 5% collagenase type II and 40 µg/ml deoxyribonuclease type I (DNAse I) (Worthington-Biochemical Corporation) at 37 °C in Dulbecco’s modified Eagle’s Medium/F12 medium (DMEM/F12) containing 15 µM Hepes buffer (Invitrogen) in a humidified incubator at 37 °C on a rotating MACSmix (Miltenyi Biotech) for 60–90 minutes. Dissociated stromal cells were separated from collagenase resistant epithelial cells (as clumps) using a 40 µm sieve (BD Bioscience-Durham) and red blood cells removed by density gradient centrifugation using Ficoll-Paque (GE Healthcare Bioscience-Bio-Sciences AB) as previously described^44^. Single cell suspensions of stromal cells were incubated with PE-conjugated SUSD2 (formerly W5C5) antibody (2 µg/ml) (Biolegend) for 30 minutes at 4 °C followed by incubation with anti-PE labelled magnetic beads (Miltenyi Biotec) for 20 minutes and SUSD2^+^ eMSC were selected using a column and magnet (Miltenyi Biotec). SUSD2^+^ eMSCs were cultured in DMEM/F12 medium containing 10% Fetal Calf Serum (FCS) (Invitrogen), 1% antibiotic-antimycotic (Life Technologies) and 2 mM glutamine (Life Technologies) for 2–4 passages. We have previously shown that this protocol isolates eMSC which robustly express the typical MSC markers when cultured (ie CD29, CD44, CD73, CD90, CD105 > 90%; CD31, CD34, CD45 < 5%).

### mCherry Lentivirus Transduction of eMSC

To detect eMSC *in vivo*, the cells were transduced with a mCherry lentivirus. Lentivirus was generated by co-transfection of three plasmids; 10 µg pLVX-IRES-mCherry (lentivirus plasmid which contains mCherry gene) (clontech-6312237), packaging plasmids; 9 µg pSPAX2 (which encodes capsid) (Addgene 12260) and 1 µg pMD2.G (encodes reverse transcriptase for lentivirus replication) (Addgene 12259), into 293 cells. (https://www.addgene.org/protocols/bacterial-transformation/) using the TransIT-X2 (Mirus) transfection reagent according to manufacturer’s protocols. Transfection was confirmed by monitoring mCherry expression by fluorescence microscopy. Viral containing supernatant was collected and passed through a 0.45um filter. eMSCs were grown to 70% confluence and transduced with lentiviral supernatant supplemented with polybrene (5ug/ml) (Sigma hexadimethrine bromide, catalogue #107689). Viral supernatant was replenished after 6 hours.

### mCherry^+^ / SUSD2^+^ eMSC Sorting

Following 48–72 hours transduction, eMSC were washed with 2% FBS/PBS, trypsinized with TrypLETM (Life Technologies) and then incubated with APC-conjugated mouse anti-human SUSD2 antibody (2 µg/ml) for 30 minutes in the dark on ice. Cells co-expressing mCherry and SUSD2 were sorted using a Beckman XDP cell sorter (Beckman Coulter, Life Science, Australia) and the data were analysed using Summit Cytomation software version 5.2. Sorted cells were then cultured until confluent for seeding onto the PA + G mesh. Lentiviral transduction does not affect the expression of the classic MSC markers or SUSD2^45^.

### Fabrication of PA + G mesh and seeding with human eMSC

Polyamide (Nylon 6) meshes were warp knitted to a mass per unit area of 42 g/m^2^ using 80 μm monofilament (Wetekam) and coated with gelatin by immersing in 12% sterile porcine type A gelatin, 300 g Bloom (G1890, Sigma Aldrich) and cross-linked with 0.0125% glutaraldehyde, as previously described^46^. PA + G meshes were cut into 1 × 1 cm pieces and gamma sterilized with 25 kGy. Prior to cell seeding, mesh was coated with 10 μg/ml fibronectin for 30 minutes at 37 °C. Mesh was then seeded at a density of 125,000 mCherry-labelled SUSD2^+^ cells/cm^2^ in 30–40 μL of medium per mesh and cultured for 48–72 hours. On the day of surgery, additional eMSCs (125,000 cells/mesh) from the same cell line were added to individual mesh pieces, together with 1 mM ruthenium metal complex, (2,2′-bipyridyl) dichloro ruthenium (II) hexahydrate [Ru II(bpy)_3_]^2+^ (Sigma-Aldrich) and 20 mM sodium persulfate (SPS) (Sigma-Aldrich), to increase cell dosage to 500,000 eMSCs/mesh. The gelatin was then cross-linked using a LED dental lamp (460 nm, 1200 mW/cm^2^, 3M Epilar Free Light 2) for 30 seconds^47^. Control meshes of PA + G (fibronectin coated) without cells were incubated in culture medium only and on the day of surgery a layer of gelatin without cells was added to the mesh and similarly cross-linked using blue light.

### Implantation of eMSC-seeded PA + G Tissue Engineering Constructs

The experimental procedure and mouse husbandry was approved by Monash Medical Centre Animal Ethics Committee A (2014/03). NSG and C57BL6 mice were housed in the animal house at Monash Medical Centre according to the National Health and Medical Research Council of Australia guidelines for the care and use of laboratory animals and were provided sterile food and water under controlled environmental conditions. Mice (48 NSG and 48 C57BL6) were randomly divided into two experimental groups of 24 mice/group and implanted with PA + G mesh seeded with (eMSC/mesh) or without (mesh control) eMSC. The mice were anaesthetized with 3% w/v Isoflurane^®^ and carprofen (5 mg/kg body weight) was used as analgesia. The abdomen was shaved and disinfected with 70% ethanol. A longitudinal 1.2 cm skin incision was performed in the lower abdomen and the skin was separated from the fascia by blunt dissection to make two pockets on each side of the midline. Tissue engineering constructs were implanted into two pockets of each animal, which received either eMSC-seeded or unseeded meshes. Meshes were sutured to the abdominal fascial layer using Dysilk 4–0^®^ sutures (Dynek) on two ends. Skin closure was performed with a single intracutaneous Dysilk 4–0^®^ suture. Animals were euthanized in a CO_2_ chamber and tissue harvested at 3, 7, 14 and 30 days (6 mice/group/timepoint using the 6 different eMSC cell lines/group of mice for each time point). Explanted meshes were dissected and divided into 3 parts for immunofluorescence, quantitative RT-PCR and ELISA.

### Immunofluorescence

Paraformaldehyde (4% for 24 hours at 4 °C) fixed tissue samples frozen in OCT were cut (8 µm) and stained with rat anti-mouse F4/80 antibody (Table 1) to quantify total macrophages, rabbit anti-mouse CCR7 for pro-inflammatory M1 macrophages and rat anti-mouse CD206 for anti-inflammatory M2 macrophages (Table 1). Sections were thawed and blocked with protein block (Dako) for 1 hour at RT. After one wash in PBS, primary antibodies were incubated for 1 hour at RT. Isotype-matched antibodies (rat IgG2b, rat IgG2a and rabbit monoclonal IgG) were used as negative controls and applied at the same concentrations. After washing, Alexa-Fluor-488 and Alexa-Fluor-568-conjugated secondary antibodies were incubated for 30 minutes at RT, respectively. Nuclei were stained with Hoechst 33258 (Molecular Probes) for 3 minutes and the slides were mounted with fluorescent mounting medium (Dako). To avoid the overlap of the red Alexa Fluor 568 and mCherry, all the slides used for CCR7 immunostaining were first checked for the presence of mCherry signal and those containing mCherry^+^ cells were excluded from the immunostaining.

**Table 1:** Details of antibodies used in immunostaining.

Primary antibody	Isotype	Supplier	Dilution(Concentration)	Secondary Ab	Concentration	Supplier
CCR7	Rabbit monoclonal	Abcam	1/200 (1 µg/ml)	Donkey anti rabbit IgG	1/500	Life Technologies
CD206	Rat IgG2a- Alexa Fluor 647 conjugated	Biolegend	1/100 (5 µg/ml)	—	—	—
F4/80	Rat IgG2b	BioRad	1/200 (1 µg/ml)	Chicken anti rat IgG	1/500	Life Technologies

### Image analysis

Four images were taken per stained section from one frozen block at each time-point using a FV1200 confocal microscope (Olympus, Life Science) at 20 × magnification. Mesh knots and the gelatin layer were included and images were taken from the center of the mesh avoiding the sutures. The images were analyzed using Image J software (National Institute of Health, NIH)^48^ to measure positive signals around mesh filaments in 100 μm increments, as described previously^49^. The percentage of M1 (red) and M2 (white) macrophage immunostaining was calculated as a proportion of total macrophages (green), co-localised in the same sections. Cell numbers were calculated as a ratio of co-localized cells (green-red-blue) and/or (green-white-blue) to total macrophages (green-blue).

### Lysate preparation and protein assay

The frozen collected tissues were thawed and cut in half and 300 µl of lysis buffer^50^ was added and homogenized on ice for one min using a homogenizer (IKA T 10 basic ULTRA-TURRAX). The supernatant was collected following centrifugation at 13,000 rpm for 10 minutes. Then 3 µl of the supernatant was used to quantify total protein content using a bicinchoninic acid assay (BCA) according to the manufacturer’s directions (Thermo Scientific) and read at 562 nm using a plate reader (Biotek). Lysate were stored in −80 °C prior to use in ELISAs.

### ELISAs

The inflammatory cytokines, Il-1β and Tnf-α, were assessed by ELISA using mouse commercial kits (BD Bioscience OptEIA). The plates were coated with capture antibodies and incubated overnight at 4 °C. The following day the plates were blocked with assay diluent (10% FBS in PBS) for one hour. The samples were diluted 1/10 in assay diluent and 50 µl of each of the samples and standards were loaded into the wells of 96 well plates. After 2 hours incubation at room temperature (RT), the plates were washed three times and incubated with biotinylated detection antibody for 1 hour followed with washing; then streptavidin-HRP was added to each well and incubated for 30 minutes at RT. Finally, a stop solution was added and the plates were read at 450 nm using a plate reader (Biotek). The cytokine concentration of the samples was obtained from the four-parameter logistic standard curve and normalized to total protein for each sample.

### qRT-PCR

RNA was extracted from explanted tissue using a RNA extraction mini kit (Qiagen-Life Technologies) according to manufacturer’s protocol and the quality and yield determined by 260/280 and 230/260 absorbance in a Nanodrop (Lab Gear). The extracted RNA was stored at −80 °C until use. First-strand cDNA was synthesized using SuperScript III first-strand synthesis system (Invitrogen). 100 ng of cDNA was then measured and assessed by quantitative PCR. M2 macrophage markers including Mannose receptor, Arginase and *Il10* mRNA and M1 macrophage markers including *Il1b* and *Tnfa* expression using SYBR Green Super Mix. Primer sets are detailed in Table 2. The PCR conditions consisted of initial denaturation at 95 °C for 10 minutes, followed by 40 cycles of denaturation at 95 °C for 15 seconds and annealing/polymerization at 60 °C for 60 seconds. To determine the relative expression of target mRNA, the expression level of each gene was normalized to the expression level of GAPDH mRNA as an endogenous control and relative expression was reported as ∆Ct.

**Table 2:** Primer sequences.

Primer sequences	Forward	Reverse
Arginase I (*ArgI*)	5′ CATGAGCTCGCCAAAGT 3′	5′ TTTTTCCAGCAGACCAGCTT 3′
Mannose Receptor (*Mrc1*)	5′ TGGCATGTCCTGGAATGAT 3′	5′ CAGGTGTGGGCTCAGGTAGT 3′
Interleukin 10 (*Il10*)	5′ TTTGAATTCCCTGGGTGAGA 3′	5′ AGACACCTTGGTCTTGGAGC 3′
*Il1b*	5′ TCACCCAAGACTCTGCCTTTAC 3′	5′ CCATGGTGGCAGTGAGGTTT 3′
*Tnfa*	5′ CCCTCACACTCAGATCATCTTCT 3′	5′ GCTACGACGTGGGCTACAG 3′
*GAPDH*	5′ AACTTTGGCATTGTGGAAGG 3′	5′ ACACATTGGGGGTAGGAACA 3′

### Statistics

Statistical analysis was performed using GraphPad Prism v7. Data were normally distributed and analyzed with two-way ANOVA (comparison of eMSC/mesh vs mesh control and time-points) and three-way ANOVA (comparison of eMSC/mesh vs mesh control, four time-points and two mouse models) both followed by Tukey posthoc test. Data are presented as mean +/− SEM.

## Electronic supplementary material

H&E staining of explanted tissue at day 14 and 30.

M2/M1 ratio in eMSC/mesh and mesh control groups
