# Supplementary material for: Endometrial Mesenchymal Stem/Stromal Cells Modulate the Macrophage Response to Implanted Polyamide/Gelatin Composite Mesh in Immunocompromised and Immunocompetent Mice
Source: Sci Rep. 2018 Apr 26;8:6554. doi: 10.1038/s41598-018-24919-6 (PMC5919927; doi:10.1038/s41598-018-24919-6)
Supplement: Supplementary file 1 — H&E staining of explanted tissue at day 14 and 30. [file 41598_2018_24919_MOESM1_ESM.pdf]

Endometrial Mesenchymal Stem/Stromal Cells Modulate the Macrophage Response to Implanted Polyamide/Gelatin Composite Mesh in Immunocompromised and Immunocompetent Mice

Darzi S[a,b], Deane JA[a,b], Nold CA[a], Edwards S[c] , Gough DJ[a], Mukherjee S[a],Gurung S[a,b],Tan KS[a], Vashi AV[c], Werkmeister JA[a,b,c], Gargett CE[a,b*]

a. The Ritchie Centre, Hudson Institute of Medical Research, 27–31 Wright Street, Clayton, Victoria 3168, Australia
b. Department of Obstetrics and Gynaecology, Monash University, Clayton, Victoria 3168, Australia,
c. CSIRO Manufacturing, Bayview Avenue, Clayton, Victoria 3169, Australia

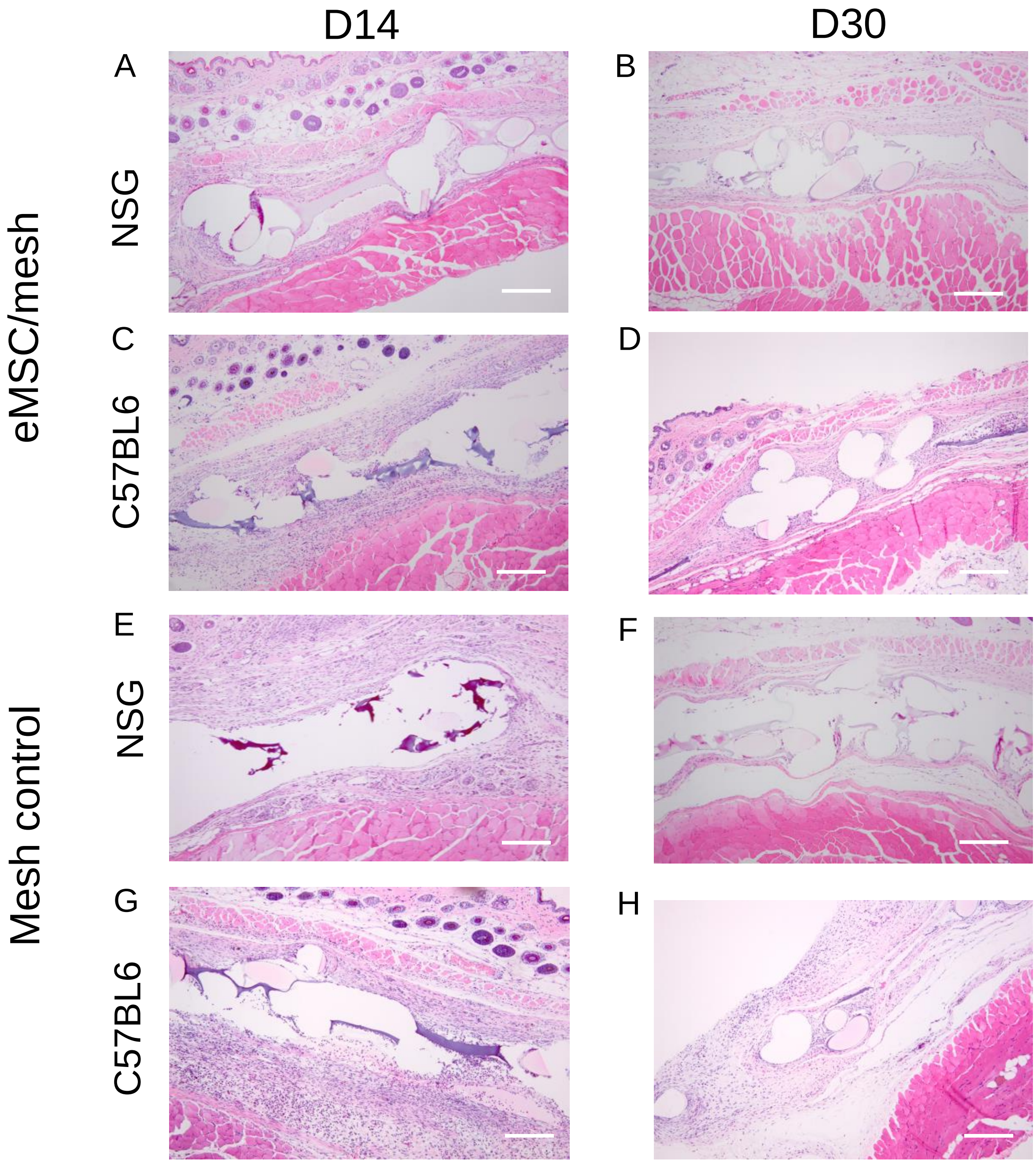

Supplementary Figure 1. H&E staining of explanted tissue at day 14 and 30.

(A, B) H&E staining on explanted tissue from NSG mice and (C, D) from C57BL6 mice, implanted with eMSC/mesh. (E, F) H&E staining on explanted tissue from NSG mice and (G, H) from C57BL6 mice implanted with mesh only. Scale Bars 200 µm.
