# Supplementary material for: Endometrial Mesenchymal Stem/Stromal Cells Modulate the Macrophage Response to Implanted Polyamide/Gelatin Composite Mesh in Immunocompromised and Immunocompetent Mice
Source: Sci Rep. 2018 Apr 26;8:6554. doi: 10.1038/s41598-018-24919-6 (PMC5919927; doi:10.1038/s41598-018-24919-6)
Supplement: Supplementary file 2 — M2/M1 ratio in eMSC/mesh and mesh control groups [file 41598_2018_24919_MOESM2_ESM.pdf]

Endometrial Mesenchymal Stem/Stromal Cells Modulate the Macrophage Response to Implanted Polyamide/Gelatin Composite Mesh in Immunocompromised and Immunocompetent Mice

Darzi S[a,b], Deane JA[a,b], Nold CA[a], Edwards S[c] , Gough DJ[a], Mukherjee S[a],Gurung S[a,b],Tan KS[a], Vashi AV[c], Werkmeister JA[a,b,c], Gargett CE[a,b*]

a. The Ritchie Centre, Hudson Institute of Medical Research, 27–31 Wright Street, Clayton, Victoria 3168, Australia
b. Department of Obstetrics and Gynaecology, Monash University, Clayton, Victoria 3168, Australia,
c. CSIRO Manufacturing, Bayview Avenue, Clayton, Victoria 3169, Australia

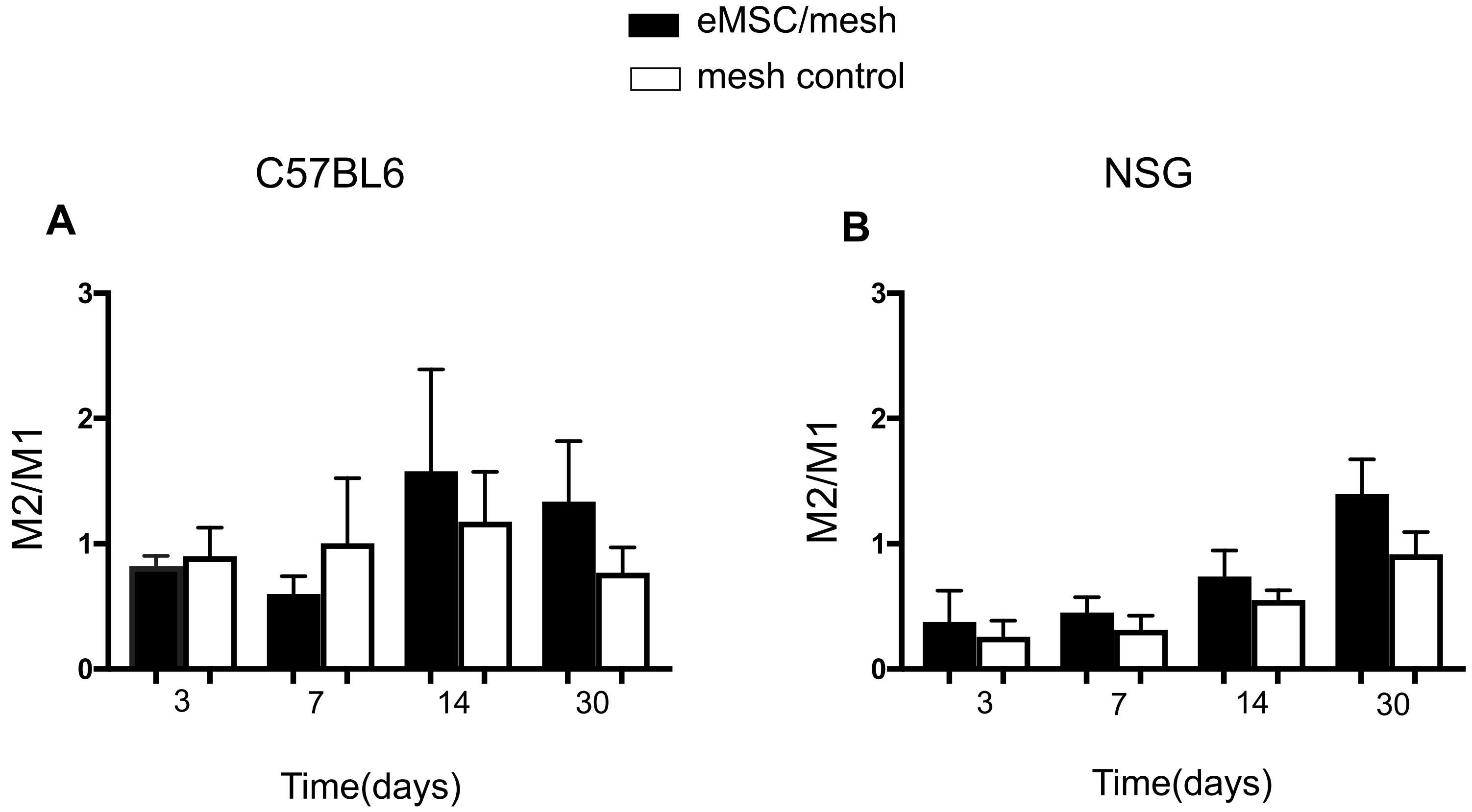

Supplementary Figure 2. M2/M1 ratio in eMSC/mesh and mesh control groups. (A) C57BL6 and (B) NSG mice
